# Haloadaptative Responses of *Aspergillus sydowii* to Extreme Water Deprivation: Morphology, Compatible Solutes, and Oxidative Stress at NaCl Saturation

**DOI:** 10.3390/jof6040316

**Published:** 2020-11-27

**Authors:** Irina Jiménez-Gómez, Gisell Valdés-Muñoz, Tonatiuh Moreno-Perlin, Rosa R. Mouriño-Pérez, María del Rayo Sánchez-Carbente, Jorge Luis Folch-Mallol, Yordanis Pérez-Llano, Nina Gunde-Cimerman, Nilda del C. Sánchez, Ramón Alberto Batista-García

**Affiliations:** 1 Centro de Investigación en Dinámica Celular, Instituto de Investigación en Ciencias Básicas y Aplicadas, Universidad Autónoma del Estado de Morelos, Cuernavaca 62209, Morelos, Mexico; irinajimenez1987@gmail.com (I.J.-G.); gisellevaldes38@gmail.com (G.V.-M.); tona.mopet@gmail.com (T.M.-P.); yordanis.perezllano@yahoo.com (Y.P.-L.); nildita1985@gmail.com (N.d.C.S.); 2 Centro de Investigación Científica y de Educación Superior de Ensenada, Ensenada 22860, Baja California, Mexico; rmourino@cicese.mx; 3 Centro de Investigación en Biotecnología, Universidad Autónoma del Estado de Morelos, Cuernavaca 62209, Morelos, Mexico; maria.sanchez@uaem.mx (M.d.R.S.-C.); jordi@uaem.mx (J.L.F.-M.); 4 Department of Biology, Biotechnical Faculty, University of Ljubljana, 1000 Ljubljana, Slovenia; nina.gunde-cimerman@bf.uni-lj.si; 5 Centro de Ciencias Genómicas, Universidad Nacional Autónoma de México, Cuernavaca 62209, Morelos, Mexico

**Keywords:** *Aspergillus sydowii*, halophily, extremophilic fungi, saline stress, saturated NaCl solution, water deprivation, oxidative stress, morphology

## Abstract

Water activity (a_w_) is critical for microbial growth, as it is severely restricted at a_w_ < 0.90. Saturating NaCl concentrations (~5.0 M) induce extreme water deprivation (a_w_ ≅ 0.75) and cellular stress responses. Halophilic fungi have cellular adaptations that enable osmotic balance and ionic/oxidative stress prevention to grow at high salinity. Here we studied the morphology, osmolyte synthesis, and oxidative stress defenses of the halophile *Aspergillus sydowii* EXF-12860 at 1.0 M and 5.13 M NaCl. Colony growth, pigmentation, exudate, and spore production were inhibited at NaCl-saturated media. Additionally, hyphae showed unpolarized growth, lower diameter, and increased septation, multicellularity and branching compared to optimal NaCl concentration. Trehalose, mannitol, arabitol, erythritol, and glycerol were produced in the presence of both 1.0 M and 5.13 M NaCl. Exposing *A. sydowii* cells to 5.13 M NaCl resulted in oxidative stress evidenced by an increase in antioxidant enzymes and lipid peroxidation biomarkers. Also, genes involved in cellular antioxidant defense systems were upregulated. This is the most comprehensive study that investigates the micromorphology and the adaptative cellular response of different non-enzymatic and enzymatic oxidative stress biomarkers in halophilic filamentous fungi.

## 1. Introduction

Water activity (a_w_) is a thermodynamic descriptor that indicates the amount of free water in an aqueous solution [1] and one of the most important factors that limit microbial growth [2,3,4]. Dissolved sodium chloride reduces a_w_ and acts as a potent cellular stressor. It entropically promotes supramolecular disorder, osmotic stress, oxidative damage, and cytotoxicity [4,5], therefore microbial life at hypersaline conditions requires extensive cell reprogramming [5,6,7,8,9,10].

Fungi display a variety of adaptations to water deprivation induced by high NaCl concentrations [6]. For example, hypersaline conditions promote in extremely halotolerant *Hortea werneckii* and halophilic *Wallemia ichthyophaga* the production of pigments, meristematic growth, synthesis of compatible solutes, modifications of the cell wall ultrastructure and fluidity of the cell membrane, upregulation of certain membrane transporters, and responsiveness of the MAPK signal-transduction system, among others [6,10]. *H. werneckii* and *W. ichthyophaga* have been widely studied as model halophilic fungi. *H. werneckii* can grow from 0% to 35% NaCl (saturation a_w_ ≅ 0.75), whereas *W. ichthyophaga* is an obligated halophile that thrives from 10% to 35% NaCl [6].

Genus *Aspergillus* contains several species well adapted to low a_w_ induced by NaCl, however it has been so far poorly studied for haloadaptative abilities. To date, the osmoadaptation mechanisms have been investigated in *A. cristatus* [11], *A. montevidensis* [12], *A. ruber* (previously *Eurotium rubrum*) [7], *A. salisburgensis*, and *A. sclerotiales* [13]. In this study we focused on haloadaptative strategies of *A. sydowii*, an important pathogen of coral reefs, with a potential reservoir in hypersaline environments. Previously, we analyzed the transcriptional response of *A. sydowii* at 0.5, 1.0 and 2.0 M of NaCl [14]. In this study we investigated the morphological and physiological responses, the synthesis of compatible solutes, and the oxidative balance of *A. sydowii* EXF-12860, grown in 1.0 M and 5.13 M (saturated) NaCl solution. We also analyzed the transcriptional expression of genes involved in the redox balance and in the cellular responses to oxidative stress caused by hypersaline conditions. To the best of our knowledge, this is the first study that evaluates oxidative stress caused by low a_w_ due to high NaCl in a halophilic fungus.

## 2. Materials and Methods

### 2.1. Strain, Preservation, and Culture Conditions

*A. sydowii* EXF-12860 used in this study is a halophilic fungus isolated from solid fermentation of sugarcane bagasse in the presence of 2.0 M NaCl. EXF-12860 showed optimal growth in culture media supplemented with 0.5–1.0 M NaCl [15]. The fungus was grown and maintained on Yeast Malt Agar (YMA): malt extract 10 g/L, yeast extract 4 g/L, dextrose 4 g/L, mycological peptone 5 g/L, agar 15 g/L. Spores and mycelium were preserved in 20% glycerol at −80 °C and deposited in the Ex Microbial Culture Collection of the Infrastructural Centre Mycosmo, Department of Biology, Biotechnical Faculty, University of Ljubljana (Slovenia). Cultures supplemented with two NaCl concentrations were used in this study: 1.0 M (optimal growth, a_w_ = 0.98) and 5.13 M NaCl (saturated NaCl solution, a_w_ = 0.75). Mycelium obtained from fresh cultures of EXF-12860 supplemented with 1.0 M NaCl was used as pre-inoculum in all experiments.

### 2.2. Morphological Analysis of Aspergillus sydowii EXF-12860 at Saturated NaCl Solution

The morphological characteristics of the fungal colony was examined by inoculating 0.1 g of fresh mycelium on YMA medium plates with added NaCl at 0, 1.0, or 5.13 M as final concentration. Ten-day-old cultures of *A. sydowii* EXF-12860 incubated at 28 °C in darkness were used to describe the macro-morphological appearance of the colonies. The “inverted agar block” method [16] was used for micro-morphological observations of EXF-12860, using a live-cell imaging system equipped with an inverted laser scanning microscope (NiKon Eclipse Ti-U, Tokio, Japan) with an Apo 60x/1.49 A.N. objective. A Hamamatsu Orca Flash 4.0 camera was used for data acquisition. Confocal images were analyzed quantitatively using the ImageJ software [17]. For micro-morphological examination, vegetative hyphae were fixed and stained with calcofluor white and DAPI (4′,6-diamidino-2-phenylindole). Samples were excited using a mercury lamp equipped with ET-DAPI filters. Length of the apical hyphal compartment, septation frequency, branching and nucleus indexes, and hyphal diameters were determined by analyzing 30 independent hyphae. Figures were processed and produced using Adobe Photoshop CS6 Extended (Adobe Systems Inc., San Jose, CA, USA). Representative microscopic fields are shown as final images.

### 2.3. Osmolytes in Aspergillus sydowii EXF-12860 Exposed to Saturated NaCl Concentration

The concentration of arabitol, erythritol, galactitol, glycerol, maltitol, mannitol, ribitol, sorbitol, trehalose, and xylitol was determined from 100 mg of mycelium collected by centrifugation after 0, 18, 96, and 168 h of the fungal growth in Yeast Malt Broth (YMB). Osmolytes were also determined after hypo-osmotic shocks using 200 mg of mycelium: (i) from 1.0 M to 0 M NaCl and (ii) from 5.13 M to 1.0 M NaCl (see Figure 3D). Seven-day-old fungal cultures were grown in 250 mL Erlenmeyer flasks with 100 mL YMB with either 1.0 M or 5.13 M NaCl. After mycelium was exposed to hypo-osmotic shocks, it was incubated at 28 °C for 30 min and was collected then by centrifugation.

The extraction of the compatible solutes was performed as previously described by [18]. Mycelium was dried at 60 °C and mixed with 1.5 mL of Bligh and Dyer solution (methanol:chloroform:water, 10:5:4) for 30 min. Later, chloroform (450 µL) and demineralized water (450 µL) were added. The resulting solution was vigorously shaken for 30 min, and finally centrifugated at 6000× *g* for 10 min for phase separation. One hundred microliters from the upper phase (methanol:water) were collected and 50 µL were injected into high-performance liquid chromatographer (HPLC) (Hewlett-Packard, Palo Alto, CA, USA). The chromatographic analysis was performed in an isocratic system equipped with an AMINEX-HPX87H column—300 mm × 7.8 mm—(Bio-Rad, Munich, Germany). Sulfuric acid (5 mM) was used as the mobile phase at a flow rate of 1 mL/min. Calibration curves were obtained using 5 mg/mL and 10 mg/mL analytical standards of each tested osmolytes (Sigma Aldrich, Saint Louis, MO, USA). Chromatograms were processed using ChromQuest software version 2.51 (Thermo Fisher Scientific, Waltham, MA, USA). Chromatographic analysis (*n* = 3) was performed at the Instituto de Biotecnología, Universidad Nacional Autónoma de México, Campus Morelos (Mexico).

### 2.4. Oxidative Stress Biomarkers in Aspergillus sydowii EXF-12860 at Hypersaline Conditions

Supernatants and cell fractions were obtained by centrifugation at 6000× *g* after 96 h of fungal growth in 250 mL of YMB media with 1.0 M or 5.13 M NaCl. Cells were ground in liquid nitrogen and 1 mL milliQ H_2_O was added per sample. Determinations were performed from both supernatants and cell fractions (*n* = 3).

Three antioxidant biomarkers were determined. Superoxide dismutase activity was evaluated by measuring the inhibition of pyrogallol autoxidation [19]. Briefly, 32 μL of a 7.37 mM pyrogallol solution in distilled water was mixed with Tris-HCl buffer (50 mM, pH 8.2) and 0.6 mM ethylenediaminetetraacetic acid (EDTA). After adding 10 μL of sample, the rate of oxidized pyrogallol (purpurogallin–quinone) formation was measured spectrophotometrically at 405 nm during 1 min [20]. As well, catalase activity was determined using EnzyChromTM Catalase Assay kit (BioAssay System, Hayward, CA, USA) and glutathione peroxidase was monitored using Glutathione Peroxidase Assay kit (Abcam, Boston, MA, USA). Finally, the concentration of reduced glutathione was quantified as described previously by [21] with minor modifications. Briefly, 5 μL of the sample was mixed with 0.1 M phosphate buffer pH 8 and 50 mL of 10^−2^ M 5′-dithiobis-(2-nitrobenzoic acid) (DNTB). The production of color due to the DNTB oxidation was measured spectrophotometrically at 412 nm. The concentration of reduced glutathione was determined using a standard curve of reduced glutathione prepared in 0.1 M phosphate buffer pH 8.

Additionally, five oxidation biomarkers were analyzed. The concentration of hydrogen peroxide was measured using BIOXYTECH^®^ H2O2-560 Assay kit (Bioxytech, Portland, OR, USA). Advanced oxidation protein products were quantified as previously described [22]. As another oxidation indicator, a colorimetric assay for lipid peroxidation markers was performed. The levels of malondialdehyde and 4-hydroxyalkenals were determined at 586 nm according to [23]. Finally, lipid peroxidation susceptibility was determined as reported by [24]. Samples were incubated with 2 mM copper sulphate at 37 °C for 24 h. Malondialdehyde levels were measured spectrophotometrically at 586 nm.

### 2.5. Transcriptomic Expression of Genes Involved in Cellular Oxidative Stress Defenses

A transcriptomic dataset of *A. sydowii* EXF-12860 growing at 1.0 M and 5.13 M NaCl was analyzed to identify differentially expressed genes related to cellular redox balance and oxidative stress responses at hypersaline conditions. This dataset is publicly available at the National Centre for Biotechnology Information (NCBI): Submission ID: SUB8102769, BioProject PRJNA662826, BioSample accession: SAMN16095160. For this experiment, total RNA was isolated from mycelium samples after 96 h of fungal growth in media with 1.0 M and 5.13 M NaCl. Transcriptomes (*n* = 3) were sequenced by Macrogene Company (Seoul, Korea) and analyzed as previously described by [14]. Sequencing quality control was performed using Trimmomatic version 0.39 [25] and Rcorrector was conducted to correct Illumina sequencing errors [26]. Trinity version 2.10.0 was used as *de novo* assembly algorithm [27]. Functional annotations and analysis of the transcriptional levels of genes related to redox balance and oxidative stress responses were performed using Blast2GO (Biobam Bioinformatic, 2019).

### 2.6. Statistical Calculations

All experimental determinations were performed in triplicates with three technical replicates. Statistical differences (*p* ≤ 0.05) among the mean of morphological determinations or the amounts of oxidative stress markers were determined by one-way ANOVA and Tukey’s HSD test as post hoc analysis. Differences (*p* ≤ 0.05) among the mean amounts of osmolytes were determined by Kruskal-Wallis test, and Dunn’s test as post hoc analysis. Statistical calculations were performed using GraphPad version Prism8 (https://www.graphpad.com). Non-metric multidimensional scaling (NMDS) analysis to visualize statistical ordinations of data from the osmolyte determinations, was performed in R using vegan package (R Development Core Team).

To analyze the grouping of samples according to oxidative stress biomarker levels we performed a principal component analysis (PCA) using the PCA function of the FactoMineR package for R (R Development Core Team). Missing values were previously imputed using the regularised iterative PCA algorithm of the missMDA package for R (R Development Core Team). In both analyses, the variables were scaled to unit variance. The Pearson correlation coefficient was calculated for each pair of indicators to analyze the correlation among the different oxidative stress biomarkers.

## 3. Results and Discussion

### 3.1. Morphology of Aspergillus sydowii EXF-12860 at Different NaCl Concentrations

**Figure 1:**
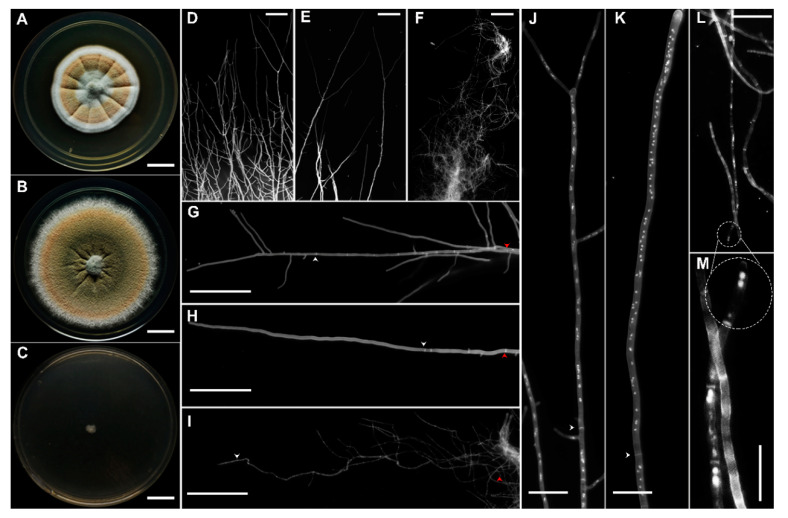
Morphological characterization of *Aspergillus sydowii* EXF-12860 grown on Yeast Malt Agar at different NaCl concentrations after 10 days of culture at 28 °C. (**A**–**C**) Colony appearance at 0, 1.0, and 5.13 M NaCl, respectively. Bars: 1 cm. (**D**–**F**) Stereoscopic examination of colonies contour after fungal growth at 0, 1.0, and 5.13 M NaCl, respectively. Bars: 100 µm. (**G**–**I**) Hyphae grown in the presence of 0, 1.0, and 5.13 M NaCl, respectively. Bars: 100 µm. White arrows indicate the first septum, while red arrows show 500 µm of length. Calcofluor white staining was applied in (**D**–**I**). (**J**–**L**) Multinucleation at 0, 1.0, and 5.13 M NaCl, respectively. Bars: 25 µm. (**L-M**) Small and large circles show the apical compartment of the hypha grown in 5.13 M NaCl. (**M**) A close-up of L. Bars: 25 µm. Staining using calcofluor white and DAPI was applied in (**J**–**M**).

The micro- and macro-morphological characters of *A. sydowii* at 0, 1.0, and 5.13 M NaCl were examined using 10-day-old cultures incubated in the dark (Figure 1A–M). In the absence of NaCl umbonate colonies reached 32–35 mm in diameter, they had delicately filiform contours and produced abundant orange white to light orange conidia (Figure 1A). Surface appearance was floccose in the center and velutinous in the margins, with hyaline to white and radially wrinkled mycelium. The colony reverse was yellowish to brownish. Surface exudates were reddish-brown to dark-brown. On the YMA medium supplemented with 1.0 M NaCl the colonies were larger, broadly spreading, attaining a diameter of 42–45 mm (Figure 1B). They were less umbonate, with abundant dull green to light orange conidia. The colony surface was powdery to floccose, margins entire to delicately filiform, mycelium hyaline to white, reverse slightly yellow (Figure 1B). No exudates were observed. At saturated NaCl concentration (5.13 M) colonies were distinctly smaller (5–6 mm), flat, with hyaline mycelium, non-pigmented reverse and filiform margins (Figure 1C). No conidia or exudates were observed. Based on these observations it was concluded that optimal salinity for *A. sydowii* EXF-12860 is at 1.0 M NaCl, as shown by a higher colony diameter (Tukey’s HSD, *p* ≤ 0.05) compared with growth at 0 M and 5.13 M NaCl.

Micro-morphological examination revealed drastic influence of NaCl on colony borders (Figure 1D–F). Vegetative hyphae at both 0 M and 1.0 M NaCl displayed polarized growth (Figure 1D,E), while at 5.13 M NaCl the hyphae were aberrant, curly, and tangled (Figure 1F). Increased hyphal branching was observed in the absence of salt with some regions attaining isometric growth (Figure 1D). NaCl also affected the length of the apical hyphal compartment, the septation frequency, the branching, and nucleation indexes (Figure 1G–M). 

**Figure 2:**
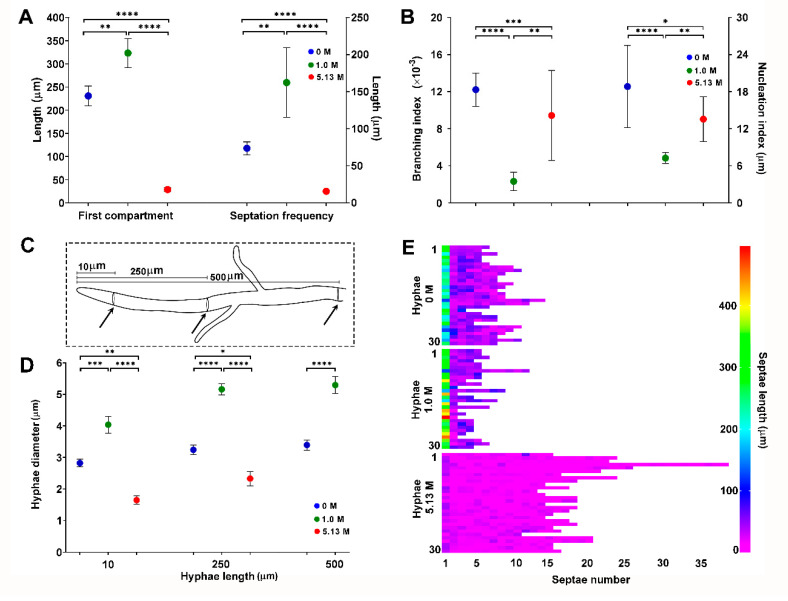
Quantification of morphological descriptors of *Aspergillus sydowii* EXF-12860 cultures grown on Yeast Malt Agar medium at 0, 1.0, and 5.13 M NaCl concentrations after 10 days of culture at 28 °C. (**A**) Length of the first (apical) hyphal compartment and septation frequency. (**B**) Branching and nucleation indexes. (**C**) Methodological representation of hyphal diameter measurements. Black arrows indicate the distances from the hyphal apex where diameters were measured. (**D**) Hyphal diameter measured at 10, 250, and 500 µm from the hyphal apex. (**E**) Septae number and septae length for 30 different hyphae. Asterisks represent statistically significant differences determined by one-way ANOVA and Tukey’s HSD test (*p* ≤ 0.05), * (*p* ≤ 0.05), ** (*p* ≤ 0.01), *** (*p* ≤ 0.001) and **** (*p* ≤ 0.0001).

The statistical analysis (Krustal-Wallis, *p* ≤ 0.05) of these morphological characters showed significant differences when *A. sydowii* EXF-12860 grew on YMA with 1.0 M and 5.13 M NaCl, or in the absence of salt (Figure 2A,B). While the average length of the apical hyphal compartment was 323 µm and 231 µm at 1.0 M and 0 M NaCl, respectively, much smaller apical compartments (29 µm on average) were observed at 5.13 M NaCl (Figure 2A). Septation was significantly increased during fungal growth at saturated NaCl solution, with one septum each 15.5 µm versus one septum each 162 µm at optimal 1.0 M NaCl condition (Figure 2A). At stress conditions (0 M and 5.13 M NaCl), also branching and nucleus indexes were markedly higher in comparison to growth at optimal salt condition (Figure 2B). The diameters of hyphae grown at different NaCl concentrations were measured at 10, 250, and 500 µm from the hyphal apex (Figure 2C). Hyphae on YMA with optimal 1.0 M NaCl had the largest diameters (Figure 2D), while the average length of hyphal compartments—distance between two septa—was smallest at 5.13 M NaCl (Figure 2E).

In summary, the micro-characterization of *A. sydowii* EXF-12860 showed drastic changes at saturated NaCl concentrations, which indicates a sharp osmotic stress. In general, EXF-12860 revealed a similar micro-morphology on YMA saturated with NaCl and in the absence of salt. Although saturated NaCl solutions significantly restrict development of most fungi [6], *A. sydowii* EXF-12860 grew even at extremely low a_w_ conditions, adapting by the formation of un-polarized, highly septated hyphae, with increased multinucleation, that so far has not been studied in halophilic fungi, and could represent an important, yet so far overlooked haloadaptation.

Besides colony morphology and pigmentation, salt stress drastically alters cell wall structure in fungi [6]. While some studies focused on the micro-structure of the cell wall of halophilic/xerophilic fungi [14,18,28], colonial micro-morphology has been rarely investigated [12,28]. Previous studies showed changes in size and appearance in colonies in the extremely halotolerant black yeast *H. werneckii* and different species of the halophilic/xerophilic basidiomycetous genus *Wallemia* [28]. Within the genus *Aspergillus*, only *A. montevidensis* was analysed. At 3.0 M NaCl it displayed unique morphological responses on the level of pigment production and cleistothecium development [12,29]. Our observations of reduced hyphal width at salt stress were in accordance with [29] and [30] for *A. montevidensis*. Thinner and shorter hyphal compartments at saturated NaCl conditions were observed in halophilic *A. sydowii* EXF-12860 as well as in halophilic *W. muriae* and *W. sebi* [28]. In mildly halotolerant *Aspergillus repens* shortening and thickening of the hyphal compartments were observed under NaCl stress compared to the non-stressed condition [31]. In an *Exophiala* sp., a recent report showed that dimorphic change was triggered by NaCl (from filamentous to yeast type phenotypes) [32].

Observed changes in hyphal geometry due to non-optimal NaCl concentrations indicate a dynamic rearrangement of the cytoskeleton in particular at 5.13 M NaCl. Septins and formins, the main proteins involved in hyphal morphogenesis, are implicated in sculpting the filamentous fungal cells [33,34], and in hyphal nuclear dynamics [35]. Our results suggest that NaCl could influence the expression of these proteins, since septation, hyphal polarity, diameter, and morphology were modified at hypersaline conditions. These proteins have been recognized previously in *Aspergillus* as key components of hyphal morphogenesis [36,37]. For example, genes encoding for septins in *A. nidulans* (*asp*A-E) have been related to the hyphal branching pattern and conidiophore development [33], while the septin orthologous genes in *A. fumigatus*, *asp*A, *asp*B, *asp*C, and *asp*E, coordinate interseptal distances in the apical and subapical hyphal compartments [38]. *Sep*A, a formin homolog of *A. nidulans*, was related to septation and hyphal diameter [33]. Although nothing has been investigated regarding septin and formin’s role in halophilic fungi, this study provides interesting initial insights into the potentially decisive influence of NaCl on their expression.

### 3.2. Synthesis of Compatible Solutes by Aspergillus sydowii EXF-12860 Exposed to NaCl

**Figure 3:**
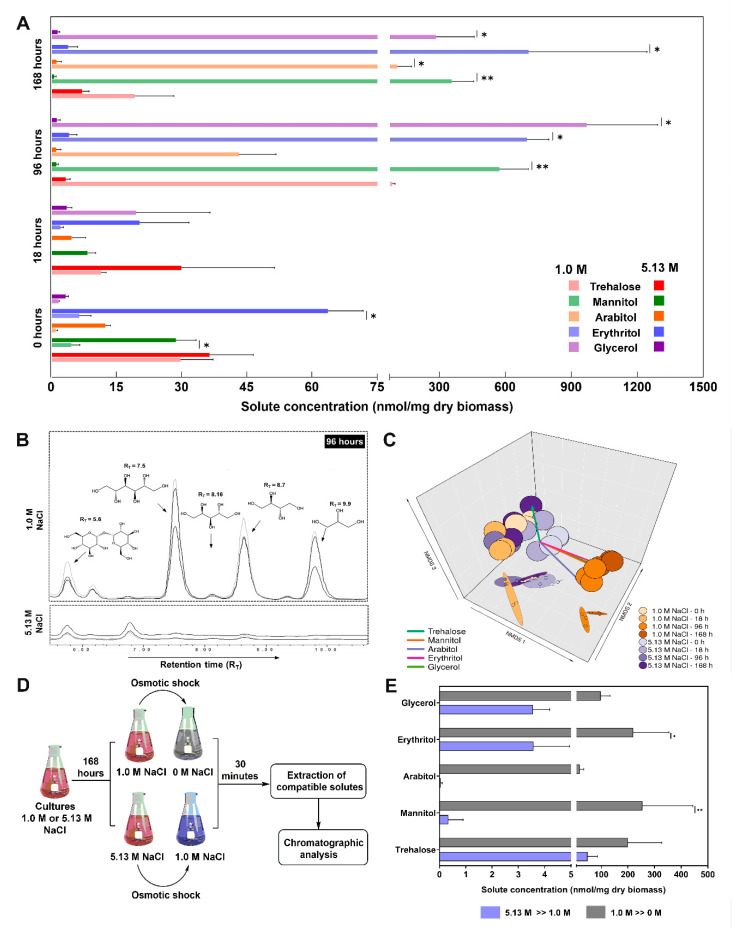
Osmolyte production by *Aspergillus sydowii* EXF-12860 grown at 1.0 M and 5.13 M NaCl (saturated NaCl solution) in Yeast Malt Broth at 28 °C. (**A**) Synthesis of compatibles solutes at 0, 18, 96 and 168 h. (**B**) Representative chromatograms obtained after 96 h of fungal culture at 1.0 M and 5.13 M NaCl. (**C**) Non-metrical multidimensional scaling (NMDS) analysis of the compatible solute production. (**D**) Experimental design to study the compatible solute production due to hypo-osmotic shocks. (**E**) Blue bars represent the compatible solute production after the hypo-osmotic shock from 5.13 M to 1.0 M NaCl. Grey bars represent the compatible solute production after the hypo-osmotic shock from 1.0 M to 0 M NaCl. Asterisks represent statistically significant differences determined by the Kruskal-Wallis test and Dunn’s test (*p* ≤ 0.05), * (*p* ≤ 0.05) and ** (*p* ≤ 0.01).

*A. sydowii* EXF-12860 produced trehalose, mannitol, arabitol, erythritol, and glycerol as compatible solutes at both tested salinities (Figure 3A,B). Initially (after 18 h), cultures growing on 5.13 M NaCl medium produced higher quantities of trehalose, mannitol, arabitol, and erythritol in comparison to older cultures (96 h and 168 h) (Figure 3A). Mannitol and arabitol were synthesized only in the NaCl saturated medium, but decreasing in time, while the glycerol concentration did not change throughout the measuring period (Figure 3A). At optimal conditions (1.0 M NaCl) the production of compatible solutes peaked at 96 h (Figure 3A), corresponding with the midpoint of the *A. sydowii* EXF-12860 exponential growth (data not shown). Arabitol peaked at 168 h (stationary growth phase) (Figure 3A). The tridimensional NMDS showed two statistical ordinations based on the responses of *A. sydowii* EXF-12860 on the level of osmolyte synthesis at different times at 1.0 M and 5.13 M NaCl (Figure 3C). Synthesis of compatible solutes at 1.0 M after 96 h and 168 h exhibited a strong ordination, reflecting these conditions as optimal physiological conditions, differing greatly from other tested experimental conditions (Figure 3C).

The compatible solute profile was determined as well after 30 min of different hypo-osmotic shocks (Figure 3D). When EXF-12860 was transferred from 5.13 M to 1.0 M NaCl trehalose was synthesized abundantly, erythritol and glycerol were produced in lesser amounts, while arabitol was not detected at all (Figure 3E). Interestingly, transfer from 1.0 M to 0 M NaCl resulted in more drastic changes, in particular mannitol considerably increased (Figure 3E). In contrast, its concentration did not change notably when *A. sydowii* was transferred from 5.13 M to 1.0 M NaCl. Arabitol displayed a similar concentration profile like mannitol.

Galactitol, maltitol, ribitol, sorbitol, and xylitol were not detectable. Figure 3B shows a properly resolved chromatographic signal that corresponds to an unknown compound eluted at 6.2 min (at 1.0 M NaCl) and another at 6.8 min (at 5.13 M NaCl) (Figure 3B). Interestingly, the concentration of this last unidentified compound increased with salinity. Since these peaks did not correspond to any analytical standards, they probably represent new osmolytes synthesized by *A. sydowii* EXF-12860.

Production of compatible solutes has been recognized as one of the main responses employed by extremophilic/extremotolerant fungi growing at low a_w_ [6]. Extremely saline conditions induce the synthesis of polyols and sugars (e.g., glycerol, erythritol, arabitol, mannitol) in halophilic and halotolerant fungi such as *H. werneckii*, *W. ichthyophaga*, and *Aureobasidium pullulans* and *Aureobasidium subglaciale*, with glycerol as the main compatible osmolyte [6,18,39,40]. Opposite to the expected, in *A. sydowii* EXF-12860 glycerol or any other polyol concentrations did not correlate positively with increased salinity (Figure 3A) and did not counterbalance the osmotic imbalances. However, synthesis of other compatible solutes increased at optimal 1.0 M NaCl (Figure 3A), reflecting physiological adaptations conveyed by these small molecules.

Our results showed growth-phase dependence of osmolyte production, as previously reported for *H. werneckii*, where glycerol was accumulated predominantly during the exponential phase and erythritol during the stationary phase [9]. Similar results were found for *Aspergillus niger* when glycerol and erythritol were determined as primary osmolytes in young mycelium, and mannitol and erythritol were predominant in older mycelium [41,42]. Interestingly, *A. sydowii* EXF-12860 steadily accumulated mannitol, erythritol, and glycerol during exponential and stationary growth phase at 1.0 M NaCl (Figure 3A), while arabitol increased also gradually during the exponential phase but peaked at stationary phase. The amounts of osmolytes remained surprisingly low at 5.13 M NaCl, with no growth-phase dependence (Figure 3A).

As observed for *W. ichthyophaga* [18,39], hypo-osmotic shocks affected the profile of compatible solutes in *A. sydowii* EXF-12860. The osmolyte profile was most different when *A. sydowii* EXF-12860 was transferred to the medium without added NaCl, suggesting a quick reprogramming during hypo-osmotic shocks and growth in a culture medium without NaCl (Figure 3E).

### 3.3. Oxidative Stress Defenses Induced by NaCl in Aspergillus sydowii EXF-12860

**Figure 4:**
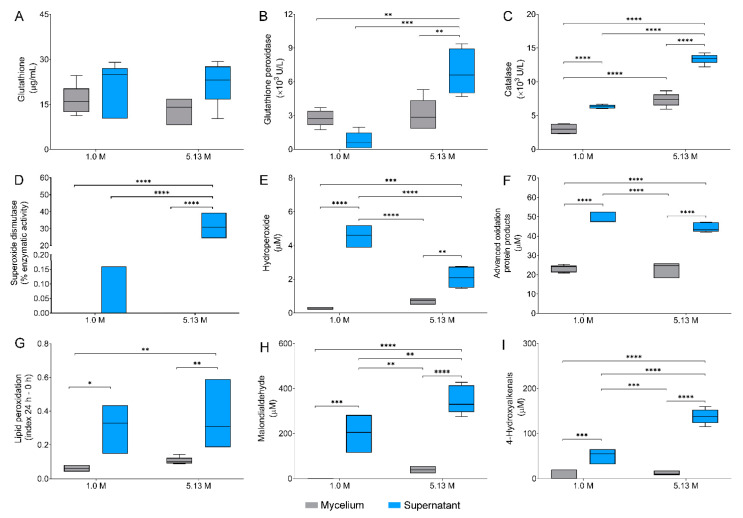
Oxidative stress responses in *Aspergillus sydowii* EXF-12860. Nine oxidative stress biomarkers were investigated in mycelium and supernatants of EXF-12860 grown at 1.0 M and 5.13 M NaCl: (**A**) Glutathione concentration; (**B**–**D**) Enzymatic activity of glutathione peroxidases, catalases, and superoxide dismutases, respectively; (**E**–**I**) Determination of hydroperoxides, advance oxidation protein products, lipid peroxidation ratio, malondialdehyde, and 4-hydroxyalkenals, respectively. Asterisks represent statistically significant differences determined by one-way ANOVA and Tukey’s HSD test (*p* ≤ 0.05), * (*p* ≤ 0.05), ** (*p* ≤ 0.01), *** (*p* ≤ 0.001) and **** (*p* ≤ 0.0001).

Different stress conditions, including water deprivation due to high concentrations of NaCl, cause oxidative damage to cells [43,44,45]. The link between the antioxidative capacity of fungi and halotolerance on the genomic level has been investigated only recently in the halophilic *W. ichthyophaga*, and halotolerant black yeasts *H. werneckii* and *A. pullulans* [43], with a single study on this subject performed previously [46]. Here we investigated the cellular antioxidant defense of *A. sydowii* EXF-12860 exposed to two NaCl concentrations: 1.0 M and 5.13 M, based on the response of nine non-enzymatic and an enzymatic biomarkers (Figure 4A–I).

The levels of reduced glutathione in mycelial fraction were similar at 1.0 M NaCl (16.40 µg/mL) and at 5.13 M NaCl (13.02 µg/mL) (Figure 4A). In both cases reduced glutathione was transported in the supernatants reaching 21.45 µg/mL and 22.18 µg/mL, without any statistical differences. Glutathione peroxidase had a considerable higher activity at 5.13 M NaCl in comparison to 1.0 M NaCl in both mycelial and supernatant fractions (Figure 4B), by 7.7 and 1.2 fold in supernatants and mycelia, respectively. Catalase and superoxide dismutase activities also increased significantly at 5.13 M NaCl (Figure 4C,D). While catalase activity increased two-fold in both mycelium and supernatants exposed at 5.13 M NaCl, superoxide dismutase increased significantly, approximately 197-fold in the supernatant at 5.13 M NaCl in contrast to optimal 1.0 M NaCl concentration. Interestingly, superoxide dismutase was not detected in mycelium at any NaCl concentration.

Additionally, five metabolic biomarkers indicative for oxidative stress were studied, due to recognized superoxide anion (O^2−^) causing damage to all major groups of biomolecules [47]. Determined concentrations of hydroperoxides were considered as direct indicators of biomolecule oxidation. Hydroperoxides were three-fold higher in the mycelium of *A. sydowii* EXF-12860 grown at 5.13 M NaCl (Figure 4E). Advanced oxidation protein products were found to the same levels at both NaCl concentrations (Figure 4F), as well as the index of lipid peroxidation of mycelia (Figure 4G). Interestingly, lipid peroxidation was detected in a high ratio in the extracellular medium at both NaCl conditions. Malondialdehyde, a naturally occurring product of lipid peroxidation [23], was detected in higher concentrations in the supernatants (Figure 4H), and only at 5.13 M NaCl also in the mycelium. Finally, 4-hydroxyalkenals, also byproducts of lipid peroxidation [23], reached the highest values in supernatants at 5.13 M NaCl (Figure 4I), but were also present as well in the mycelium at both salt concentrations.

Investigated biomarkers indicate high oxidative stress caused by NaCl saturated media (5.13 M NaCl) in *A. sydowii* EXF-12860, resulting in a robust non-enzymatic and enzymatic cellular response to overcome cellular damage (Figure 4A–D). Water deprivation by NaCl constitutes a hard-environmental stressor for eukaryotes [6,48]. Fungi respond to osmotic stress by inducing antioxidant metabolites as glutathione (non-enzymatic antioxidant response) and enzymes (enzymatic antioxidant response) as catalases, superoxide dismutases, and glutathione peroxidases, among others [43,47,49].

*A. sydowii* EXF-12860 displayed a finely regulated glutathione balance at both NaCl concentrations (Figure 4A), with no major differences between mycelia and supernatants. Glutathione is generally recognized as the most abundant antioxidant in the cell, with diminishing intracellular levels when exposed to oxidative stress [50]. An increase of the intracellular glutathione levels was also found in halotolerant bacteria cultured at high NaCl concentrations [44].

The enzymatic activities of glutathione peroxidase, catalase, and superoxide dismutase increased at 5.13 M NaCl (Figure 4B–D), indicating exposure to oxidative stress in *A. sydowii* EXF-12860. The observed enzymatic antioxidant defense contributed to the maintenance of the redox cellular homeostasis, by limiting the cellular damage caused by reactive oxygen species (ROS) [47]. ROS mainly arise as by-products of aerobic metabolism in mitochondria and include the O^2−^, hydrogen peroxide (H_2_O_2_), and hydroxyl radicals (OH·) [51]. O^2−^ must be immediately converted to H_2_O_2_ by superoxide dismutases, followed by H_2_O_2_ detoxification mediated by catalases and glutathione peroxidases. H_2_O_2_ can be also partially reduced to OH·, with an extremely high reactivity towards different biological targets [47]. Increase in ROS level activates signaling pathways to maintain the cellular redox state [47].

The oxidative damage markers studied in *A. sydowii* EXF-12860 revealed that exposure to saturated Na^+^ concentration induces damages to both proteins and lipids (Figure 4E–I). Particularly, lipid peroxidation is promoted, as shown by enhanced lipid peroxidation ratio and higher concentrations of hydroperoxides, malondialdehyde, and 4-hydroxyalkenals (Figure 4E,H,I), as well documented previously [52]. Oxidation of lipids involves continuous formation of hydroperoxides as primary oxidation products which are in turn reduced by glutathione peroxidases with glutathione as the reductant [53]. One of the end products of lipid peroxidation is the generation of malondialdehyde [52], as well as 4-hydroxyalkenals, considered as non-enzymatic peroxidation products of polyunsaturated fatty acids [54]. Increased levels of malondialdehyde were found at high salinity in the halotolerant bacteria *Planococcus* spp., *Bacillus haikouensis*, and *Microcystis aeruginosa* [44,55].

Proteins, particularly those with prosthetic Fe-S groups, are also affected by ROS [51]. Cysteine is especially susceptible, in particular the thiolate anions (Cys-S^−^) found at physiological pH [56]. For example, H_2_O_2_ mediates reversible Cys-S^−^ oxidation to sulfenic form (Cys-SOH) which triggers the oxidative stress signaling pathway. However, damaging levels of H_2_O_2_ cause irreversible Cys-S^−^ oxidation to sulfinic (SO_2_H) or sulfonic (SO_3_H) species [57], causing permanent oxidative protein damage [51]. It appears that saturated concentration of Na^+^ did not cause significant protein oxidation in *A. sydowii* EXF-12860, since the quantities of advanced protein oxidation products were similar at both salinity conditions.

Oxidative stress as a direct consequence of NaCl exposure has not been yet thoroughly investigated in halophilic/halotolareant fungi [43], as shown by the lack of reports. To the best of our knowledge this is the first study that investigates the adaptative cellular responses of different non-enzymatic and enzymatic oxidative stress biomarkers in halophilic filamentous fungi. In the extremely halotolerant black yeast *H. werneckii*, it was shown that salinity induced oxidative stress limits yeast growth [46].

**Figure 5:**
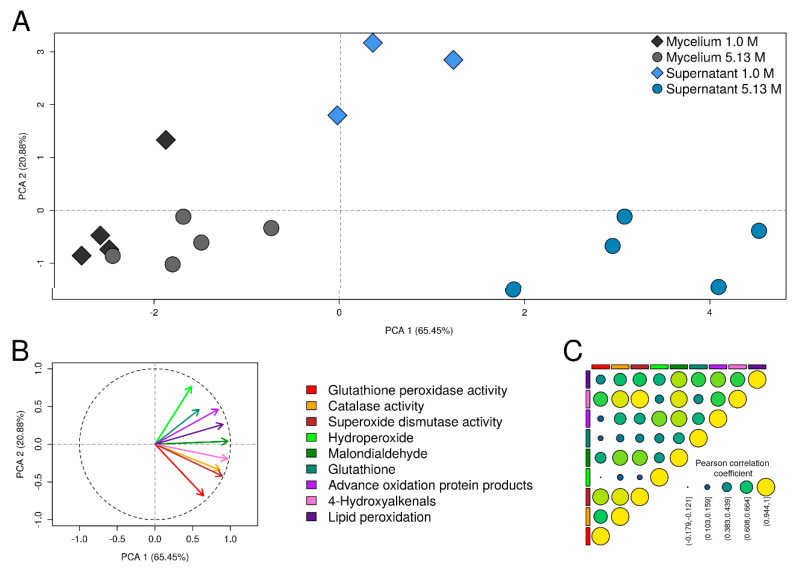
Multivariate statistical analysis of the oxidative stress responses in *Aspergillus sydowii* EXF-12860. (**A**) Principal component analysis (PCA) plot showing the grouping of mycelium and supernatant samples at 1.0 M and 5.13 M NaCl conditions according to their values of oxidative stress indicators. (**B**) The correlation between oxidative stress biomarkers and the axes of the PCA plot. (**C**) Pearson correlation coefficient of the oxidative stress biomarkers in samples of mycelium and supernatant at 1.0 M and 5.13 M NaCl conditions.

Correlation between oxidative stress biomarkers determined by PCA analysis (Figure 5A,B) showed similar variance across the oxidative stress biomarkers studied. PC1 and PC2 explained most of the variance, as expected PC1 had the largest variance (65.45%). Two potential clusters corresponding to mycelia and supernatants were identified (Figure 5A), indicating similar response of both mycelium and supernatant. Biomarkers 4-hydroxyalkenals, malondialdehyde, and lipid peroxidation ratio contributed notably to PC1, while hydroperoxide and glutathione peroxidase contributed notably to PC2 (Figure 5B). Pearson correlation analysis showed a strong correlation between 4-hydroxyalkenals, malondialdehyde, and lipid peroxidation ratio, as well as with the three antioxidant enzymes measured (Figure 5C).

In summary, *A. sydowii* EXF-12860 responded to high concentrations of NaCl with oxidative stress, shown in particular by increased levels of antioxidant enzymes and lipid peroxidation biomarkers.

### 3.4. Transcriptional Expression of Genes Related to Oxidative Stress Responses in Aspergillus sydowii EXF-12860

The transcriptome of *A. sydowii* EXF12-860 showed 44 differentially expressed transcripts involved in antioxidant responses at 5.13 M versus 1.0 M NaCl (Table 1).

While seven catalase transcripts (*cat*) were differentially expressed, only two of them were strongly upregulated (logFC = 11.27 and logFC = 10.73). All catalase transcripts corresponded to the same gene, indicating that differential transcript usage occurred in the transcriptional reprogramming of *A. sydowii* EXF-12860 at high salinity. Further, different isoforms of genes encoding for cytoplasmatic (*sod*1) and mitochondrial (*sod*2) superoxide dismutases, and cystathionine gamma-lyase (*cse*) were upregulated. These results confirmed that differential usage of alternatively spliced mRNAs took place in *A. sydowii* EXF-12860 at Na^+^ saturated concentration. Genes encoding glutathione-S-transferase (*gst*), thioredoxin (*trx*), glyoxalase (*gly*), aconitase (*aco*), cytochrome P450 (*cyp*450), and succinyl-CoA-3-ketoacid-coenzyme A transferase (*scot*) were also overexpressed. Interestingly, *oax* (logFC = 5.24) and *ish*1 (logFC = 4.84) genes that code for auxiliary antioxidant enzyme and stress response sensor respectively, were upregulated.

These results confirm that salinity stress by 5.13. M NaCl resulted in oxidative stress in *A. sydowii* EXF-12860. Thus, this halophilic fungus upregulated the transcriptional expression of different genes involved in the oxidative stress defense. Our results suggest that the transcriptional reprogramming that occurred in EXF-12860 exposed at 5.13 M NaCl could have helped to overcome the imbalance between the cellular levels of oxidants (ROS) and antioxidants. In this context, catalases, superoxide dismutases, and glutathione reductases are enzymes with antioxidant effects that limit the damage of ROS [58]. Glutathione reductases and glutathione-S-transferases play a key role in the glutathione redox cycle keeping adequate levels of reduced glutathione to connect to different ROS detoxification metabolic pathways [59,60]. Reduced glutathione also serves as a bridge with other detoxification metabolisms reacting with hydroperoxides or lipid peroxides through the glutathione peroxidase [61]. Although glutathione levels were not largely changed at 5.13 M NaCl, the overexpression of the glutathione reductase gene suggests that *A. sydowii* EXF-12860 installed a transcriptomic response to maintain reduced levels of glutathione in the fungal cell. Glutathione peroxidase was not differentially expressed, probably because the hydroperoxide levels were smaller at both NaCl concentrations (Figure 4E). This cytosolic reduced-glutathione-dependent peroxidase mediates the reduction of hydroperoxides formed in cells. Our biochemical and transcriptomic results point out that catalase, superoxide dismutase, and glutathione reductase together acted as a primary antioxidant defense system to protect *A. sydowii* EXF-12860 against peroxidant molecules at extreme salinity conditions.

Another antioxidant defense that showed upregulated genes in *A. sydowii* EXF-12860 was the thioredoxin system, which is involved in the removal of ROS, particularly H_2_O_2_. Thioredoxins sense the oxidative stress, keeping the thiol-related redox state and activate signaling proteins such as peroxiredoxins and kinases [62]. Subsequently, peroxiredoxins also play an important role in stabilizing the thioredoxin redox balance [63]. In addition, peroxiredoxins are involved in redox information signaling and controlling cell metabolism [64]. Genes encoding peroxiredoxin, thioredoxin reductase, and thioredoxin family proteins, all involved in the thioredoxin system, were upregulated in *A. sydowii* EXF-12860 grown at 5.13 M NaCl. 

Cysteine is a crucial amino acid for antioxidant cellular response [63]. In contrast, homocysteine, a methionine derivative, perturbs protein synthesis, contributes to the formation of ROS, and promotes hypermethylation reactions in the cell, which could disturb gene expression and regulation [65,66]. Thus, during oxidative stress it is crucial to efficiently reduce the levels of intracellular homocysteine which can be condensed with serine to form cystathionine by cystathionine *β*-synthase (CBS). Cystathionine is finally converted to cysteine via cystathionine gamma-lyase (CSE) [65,67]. We found that *cbs* and *cse* genes were upregulated during growth at salinity stress. As expected, homocysteine synthase genes (*cys*) were down regulated. These genes encode enzymes that catalyze the conversion of O-acetyl-L-homoserine into homocysteine in the methionine biosynthesis pathway [65]. Our results suggest that homocysteine synthesis is turned off, while the conversion of homocysteine into cysteine is transcriptionally activated. This strategy also evidences the antioxidant defense of this fungus at hypersaline conditions.

Finally, the glyoxalase system was also analyzed. The role of glyoxalases has been linked to stress response in eukaryotes contributing to confer tolerance to environmental stressors [68]. For example, they have been proposed as biomarkers for abiotic stress tolerance in plants [69]. However, glyoxalases were primarily related to the detoxification pathway of methylglyoxal, a cytotoxic product of glycolysis. Glyoxalases catalyze the conversion of methylglyoxal into D-lactate using reduced glutathione as a cofactor [70]. It has been reported that various abiotic stresses including salinity and water deprivation increase intracellular concentrations of methylglyoxal, which is removed by glyoxalases encoded by *gly*I and *gly*II genes [68]. Glyoxalase I mediates the combination of methylglyoxal with reduced glutathione to form S-lactoylglutathione, which is later converted into D-lactate via glyoxalases II that also regenerates reduced glutathione [71]. We also found upregulated *gly*I gene that encodes S-D-lactoylglutathione lyase.

Moreover, we analyzed the transcription of genes related with the lipid oxidation as it is a process strongly induced by the oxidative stress [52]. Five transcripts that corresponded to two different *scot* genes encode succinyl-CoA-3-ketoacid-coenzymeA transferase were differentially expressed (Table 1). While only one gene (transcript id: DN1270) was upregulated under salinity stress (logFC = 5.04), four isoforms of the other gene (transcript id: DN1899) were downregulated. This gene also showed a differential transcript usage. Succinyl-CoA-3-ketoacid-coenzymeA transferases are mitochondrial matrix enzymes with a central function in ketone body catabolism. The link between *scot* genes and oxidative stress has not been comprehensively studied. In the bacterium *Burkholderia pseudomallei*, it has been suggested that *scot* genes respond to external ROS [72]. Although it was hypothesized that succinyl-CoA-3-ketoacid-coenzymeA transferases are needed to transfer CoA from succinyl-CoA to oxidized lipid during oxidative stress, the mRNA levels of *scot* genes were downregulated in *B. pseudomallei*. The negative transcriptional regulation of *scot* genes may reduce the intracellular levels of acetoacetyl-CoA, and the reduction of NADH and FADH_2_, which results in smaller amount of intracellular ROS [72]. So, it is not surprising that *A. sydowii* EXF-12860 downregulated the transcription of four different transcripts encode for succinyl-CoA-3-ketoacid-coenzymeA transferases at hypersaline conditions.

Finally, *aox* gene encoding an alternative oxidase was upregulated at hypersaline condition (logFC = 5.25). Alternative oxidases, that are synthesized by a huge variety of plants, fungi, and some protists, constitute an alternative respiratory chain that confers resistance to different environmental stressors [73,74]. These enzymes allow electron flow through the oxidative phosphorylation respiratory chain even in the presence of toxic levels of ROS that inhibit complexes III and IV. The activity of alternative oxidases is crucial when occurs an accumulation of complex III-derived O_2_^−^ as a consequence of the oxidative stress [47]. It is known that *aox* genes are linked to fungal development under stress conditions [75,76]. In *Candida albicans*, alternative oxidases are intimately involved in the yeast growth during exposure to fluconazole [77], while these enzymes are relevant during the growth of *A. fumigatus* [78,79] and *Botrytis cinerea* [76] under hypoxic conditions. Additionally, *aox* genes have been upregulated in several fungi such as *A. niger*, *Yarrowia lipolytica* [80], *A. fumigatus* [81], *Hansenula anomala* [82], and *Paracoccidioides brasilenses* [83] in response to oxidative stress or water deprivation.

Our results are consistent with those reported by [46] who studied the transcriptional response in the halotolerant yeast *H. werneckii* during its growth at hypersaline conditions. In *H. werneckii*, the expression of genes encoding aconitase and Fe-S cluster protein (nicotinamide adenine dinucleotide (NADH) dehydrogenase) was increased at 17% NaCl. Besides, these genes were transcriptionally upregulated in *A. sydowii* EXF-12860 at 5.13 M NaCl. Aconitase and NADH dehydrogenase function as mitochondrial redox balance sensors and they are one of the main molecular targets of ROS [84]. At the same time, aconitase, for example, is a key enzyme in the Krebs cycle, while NADH dehydrogenase is an important component of the complex I for electron flow during mitochondrial respiration. Thus, they may be overexpressed under high salinity conditions to maintain sufficient physiological levels of enzymes keeping switched on the Krebs cycle and preserving the signaling role as a redox state sensor, or to support the ATP production, respectively [46]. Similar results were also found in the proteome of *H. werneckii* exposed to 3.0 M and 4.5 M NaCl [49].

**Figure 6:**
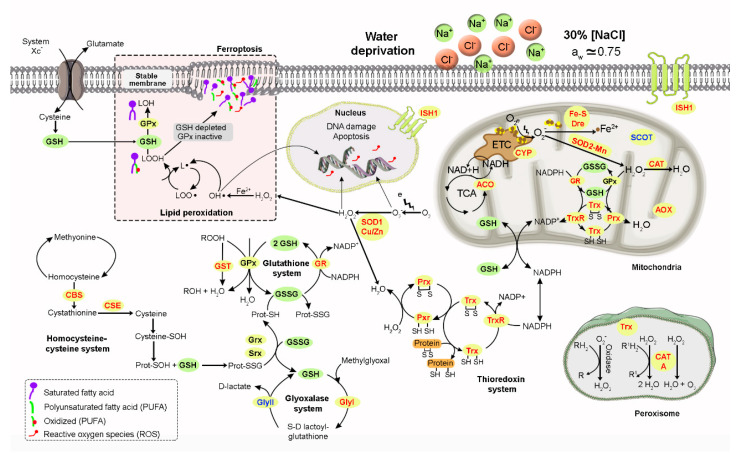
The cellular antioxidant defense model of *Aspergillus sydowii* EXF-12860 in saturated NaCl concentration (5.13 M NaCl). Green and yellow circles indicate the intermediate compounds and enzymes of the represented biochemical pathways which are involved in oxidative stress responses. Red, blue and black letters (see yellow circles) indicate upregulated, downregulated and non-differentially expressed genes, respectively.

Our biochemical and transcriptomic results point out that catalase, superoxide dismutase, and glutathione reductase together acted as a primary antioxidant defense system to protect *A. sydowii* EXF-12860 against peroxidant molecules at extreme salinity conditions. This halophilic ascomycete upregulated under Na^+^ stress a set of genes that evidences the exploitation of an extensive antioxidant response that also included the systems of glutathione, thioredoxin, and glyoxalase. Figure 6 represents a cellular model with the antioxidant defense of *A. sydowii* EXF-12860 to NaCl saturated concentration.

## 4. Conclusions

The cellular effects of water deprivation have been poorly studied in aspergilli. In this work we focused on morphology changes and osmolyte synthesis in the halophile ascomycete *Aspergillus sydowii* EXF-12860 exposed to extreme water deprivation induced by Na-saturated concentrations. We also analysed the biochemical and transcriptomic responses triggered by this fungus to overcome the oxidative stress caused by salt. Our results demonstrated that water deprivation induced by NaCl-saturated concentrations have an important impact on the cell morphology of *A. sydowii* that included an extensive morphological remolding to face salt stress. This is the most comprehensive micromorphological description in a halophilic filamentous ascomycete. *A. sydowii* produced trehalose, mannitol, arabitol, erythritol, and glycerol in the presence of both 1.0 M and 5.13 M NaCl concentrations. Interestingly, the osmolyte synthesis analyses showed that glycerol did not increase in NaCl-saturated media. Our results also support that osmolyte production exhibits a growth-phase dependence. Saturating NaCl concentrations induced oxidative stress in *A. sydowii*. Our biochemical data showed that catalase, superoxide dismutase, and glutathione reductase together acted as a primary antioxidant defense system to protect *A. sydowii* cells against peroxidant molecules at extreme salinity conditions. Reduced glutathione exhibited same levels in both 1.0 M and 5.13 M NaCl indicating that this halophilic fungus properly managed the oxidative stress caused by salty conditions. In addition, transcriptomic data revealed that genes involved in different cellular antioxidant defenses such as homocysteine-cysteine, glutathione, glyoxalase, and thioredoxin systems were upregulated. Thus, *A. sydowii* triggered an extensive antioxidant response to overcome the cellular oxidative toxicity induced by Na^+^ at extremely high concentrations. To the best of our knowledge, this is the first work describing the non-enzymatic and enzymatic antioxidant defenses in filamentous fungi exposed to hypersaline conditions. This work certainly increased our knowledge on halophilic fungi-NaCl interactions. However, new efforts are needed to fully understand the physiological reprogramming that occurred in halophilic fungi to survive in extremely salty environments.

## Figures and Tables

**Table 1:** Transcriptomic expression of *Aspergillus sydowii* EXF-12860’s genes related to oxidative stress responses induced by hypersaline condition.

Transcript ID	Annotation	Gene ID	LogFC	FDR
TRINITY_DN892_c0_g1_i12	Catalase A	*cat*A	11.27	4.46 × 10^−4^
TRINITY_DN892_c0_g1_i4			10.73	6.37 × 10^−4^
TRINITY_DN892_c0_g1_i3			−4.12	2.57 × 10^−3^
TRINITY_DN892_c0_g1_i6			−5.08	2.69 × 10^−4^
TRINITY_DN892_c0_g1_i11			−11.27	2.46 × 10^−8^
TRINITY_DN892_c0_g1_i9			−11.94	2.53 × 10^−9^
TRINITY_DN892_c0_g1_i10			−14.33	1.37 × 10^−11^
TRINITY_DN1037_c0_g1_i5	Glyoxalase I	*gly*1	11.15	4.82 × 10^−4^
TRINITY_DN1037_c0_g1_i4			10.40	9.77 × 10^−4^
TRINITY_DN2716_c0_g2_i1			3.85	5.32 × 10^−2^
TRINITY_DN1197_c0_g1_i1	Cystathionine gamma-lyase	*cth*	10.51	8.67 × 10^−4^
TRINITY_DN1197_c0_g1_i7			7.20	3.63 × 10^−6^
TRINITY_DN1197_c0_g1_i6			3.66	8.26 × 10^−3^
TRINITY_DN2112_c0_g1_i19	Cytochrome P450 alkane hydroxylase	*cyp*	9.84	9.10 × 10^−7^
TRINITY_DN3588_c0_g1_i1	Cytochrome P450	*cyp*450	6.65	4.76 × 10^−6^
TRINITY_DN2010_c0_g1_i16			4.89	3.88 × 10^−4^
TRINITY_DN2010_c0_g1_i10			4.65	7.22 × 10^−4^
TRINITY_DN312_c0_g1_i9			−4.57	1.59 × 10^−3^
TRINITY_DN312_c0_g1_i2			−3.67	8.32 × 10^−3^
TRINITY_DN4029_c0_g1_i2	Aconitate hydratase	*aco*1	8.88	7.12 × 10^−6^
TRINITY_DN1574_c0_g1_i2	Alternative oxidase-domain-containing protein	*aox*	5.25	1.47 × 10^−4^
TRINITY_DN1793_c0_g1_i1	Fe-S cluster assembly protein dre2	*dre*2	5.24	1.51 × 10^−4^
TRINITY_DN10053_c1_g1_i1	Peroxirredoxin 6	*prdx*6	5.20	1.89 × 10^−4^
TRINITY_DN2598_c0_g1_i4	Cytosolic Cu/Zn superoxide dismutase	*sod*1	5.01	2.74 × 10^−4^
TRINITY_DN2598_c0_g1_i3			4.38	1.66 × 10^−3^
TRINITY_DN1168_c0_g2_i5	Stress response protein putative	*ish*1	4.84	8.67 × 10^−4^
TRINITY_DN681_c0_g1_i14	Glutaredoxin/glutathione-dependent peroxiredoxin	*prx*1	4.73	7.03 × 10^−4^
TRINITY_DN1443_c0_g1_i3	Tiorredoxin-like protein	*trx*	4.39	1.36 × 10^−3^
TRINITY_DN1443_c0_g1_i3			−5.45	2.93 × 10^−4^
TRINITY_DN2569_c2_g2_i1	Glutathione S-transferase	*gst*	4.19	2.90 × 10^−3^
TRINITY_DN9329_c0_g1_i1			−4.15	5.47 × 10^−3^
TRINITY_DN473_c0_g1_i3			−10.53	1.67 × 10^−7^
TRINITY_DN902_c0_g1_i5	Superoxide dismutase [Mn] mitochondrial	*sod*2	4.21	3.20 × 10^−3^
TRINITY_DN902_c0_g1_i4	Superoxide dismutase Fe-Mn family	*sod*2	3.65	7.90 × 10^−3^
TRINITY_DN2375_c0_g1_i2	MAP kinase kinase kinase	*ask*1	−3.80	5.87 × 10^−3^
TRINITY_DN38_c0_g1_i5	Homocysteine synthase	*cys*D	−3.84	7.67 × 10^−3^
TRINITY_DN50_c0_g1_i16			−6.25	8.00 × 10^−5^
TRINITY_DN1899_c0_g1_i4	Succinyl-CoA:3-ketoacid-coenzyme A transferase	*scot*	−4.23	2.02 × 10^−3^
TRINITY_DN1899_c0_g1_i2			−5.14	2.47 × 10^−4^
TRINITY_DN1899_c0_g1_i5			−9.54	1.81 × 10^−6^
TRINITY_DN1899_c0_g1_i6			−9.57	1.71 × 10^−6^
TRINITY_DN298_c0_g1_i11	Glyoxalase II (Hydroxyacylglutathione hydrolase)	*gly*2	−4.45	1.78 × 10^−3^
TRINITY_DN1341_c0_g1_i2	NAD(P)H dehydrogenase (quinone)	*ipda*	−8.91	9.70 × 10^−6^
